# Nitrogen threshold under straw returning: optimizing nitrogen application to synergize the yield and nutritional stoichiometry of maize

**DOI:** 10.3389/fpls.2026.1762962

**Published:** 2026-02-25

**Authors:** Xiaolong Zhang, Xiangzeng Meng, Shan Zhang, Kaichang Liu, Yanjie Lv

**Affiliations:** 1 State Key Laboratory of Nutrient Use and Management, Shandong Academy of Agricultural Sciences, Jinan, China; 2 Maize Research Institute, Taian Academy of Agricultural Sciences, Taian, China; 3 Institute of Agricultural Resource and Environment, Jilin Academy of Agricultural Sciences, Changchun, Jilin, China

**Keywords:** no tillage with straw mulch (SM), nutritional stoichiometry, partial factor productivity of nitrogen (PFPN), plow tillage with straw mulch (SP), sustainable intensification

## Abstract

**Introduction:**

Straw returning has the potential to reduce nitrogen (N) input by enhancing soil fertility; however, the optimal N application rate may vary under different crop residue management practices.

**Methods:**

Based on a long-term field experiment initiated in 2011, this study investigated the effects of two residue management methods, namely, no tillage with straw mulch (SM, full straw return) and plow tillage incorporating straw mulch to a depth of approximately 15 cm (SP, full straw return), in combination with five nitrogen application rates (0, 90, 150, 210, and 270 kg ha^−1^) on maize grain yield, nutritional quality, and the partial factor productivity of nitrogen fertilizer (PFPN).

**Results:**

The results indicated that the 1000-kernel weight and kernel number under SP were 7.09% and 6.26% higher than those under SM, respectively, resulting in a 9.24% higher yield in SP. Furthermore, PFPN was significantly greater under SP, by 13.88% compared with SM. This difference was more evident when the N application rate was below 150 kg ha^−1^. Additionally, the comprehensive nutritional quality index (Q value), which integrates crude protein, crude fat, starch, and amino acid contents via the entropy weight method to avoid limitations of single-trait analysis, reached a higher linear plateau under SP than under SM, as SP enhanced the crude fat (by 6.68%) and starch (by 1.90%) contents in the grains.

**Discussion:**

In conclusion, SP demonstrated greater potential for N fertilizer savings while achieving high grain yield and superior nutritional quality. The optimal N application rates were 113.82–129.53 kg ha^−1^ for SP and 129.90–135.81 kg ha^−1^ for SM, ensuring the coordinated improvement of yield, nutritional quality, and PFPN.

## 1 Introduction

Maize (*Zea mays* L.), one of the world’s most important crops, provides approximately two-thirds of the global energy source for humans and animals (Simić et al., 2020). From 2000 to 2021, maize grain yield in Northeast China increased markedly by about 270%. However, achieving sustainable production that integrates both high yield and high quality remains a critical challenge in modern agricultural systems (Li et al., 2024). Nitrogen (N) is a critical factor for achieving high maize productivity; however, conventional N recommendations that prioritize yield maximization may compromise grain quality, as evidenced by diluted grain protein concentration and the insufficient enhancement of starch content relative to yield gains, while also reducing nitrogen use efficiency (NUE) and increasing production costs (Gao et al., 2023b; Guo et al., 2025; Li et al., 2025b). The widespread adoption of straw returning, a conservation tillage practice that sequesters carbon and mitigates emissions, further complicates N management (Liu et al., 2014). A central challenge for straw returning is the development of fertilization strategies that optimize nitrogen input to achieve synergistic improvements in yield and nutritional stoichiometry.

Straw returning is currently the predominant method for managing maize residues (Wang et al., 2021). Long-term straw returning enhances soil fertility and allows for reduced N input (Jiang et al., 2019). However, the decomposition rate of straw is strongly influenced by climatic factors such as temperature and soil moisture under various management practices, thereby affecting soil nutrient balance (Wang et al., 2012; Tian et al., 2019). For instance, no tillage systems with straw returning increase soil organic carbon but may cause nutrient imbalances in the C/N ratio, intensifying N competition between microorganisms and crops (Guan et al., 2020; Kan et al., 2020) and consequently decreasing grain NUE relative to plow tillage (Meng et al., 2023). In contrast, plow tillage disrupts the plow pan, facilitating improved grain yield (Kan et al., 2020; Yu et al., 2023). Appropriate N application enhances maize yield by extending the grain-filling period and increasing the mean filling rate (Wei et al., 2018; Wu et al., 2022). Some studies have reported that a 20% reduction in N application under straw returning can still maintain stable yields (Song et al., 2024). Nevertheless, factors such as latitude, climate, and cultivar variation necessitate the localization of residue management and N application strategies (Yang et al., 2022; Gao et al., 2023a; Zhang et al., 2023a). Therefore, optimizing residue management in conjunction with N fertilization is crucial for achieving high-yield, efficient maize production in Northeast China.

Grain quality is a key determinant of the economic value of maize and is markedly influenced by residue management. For example, plow tillage has been shown to increase both crude protein and starch contents in grains (Zhang et al., 2023b; Li et al., 2024). N application rate is another major determinant of grain protein concentration and starch characteristics (Wang et al., 2020; Lyu et al., 2022), although excessive fertilization can negatively affect starch properties (Chen et al., 2014). From a physiological perspective, N plays a dual regulatory role in the synthesis of grain storage protein and starch, with its effects strongly mediated by soil physical conditions altered by tillage. Plow tillage decreases soil bulk density and increases total porosity and aeration, thereby promoting root elongation (Meng et al., 2023). Enhanced root N uptake capacity ensures a continuous supply of ammonium (NH_4_^+^) and nitrate (NO_3_^−^) to the shoots, where NH_4_^+^ is preferentially assimilated into glutamine via glutamine synthetase in the leaves and subsequently transported to developing grains as a precursor for storage protein synthesis (Lee et al., 2023; Yang et al., 2025). For starch synthesis, sufficient N availability upregulates the expression of key enzymes in the grain endosperm, while improved soil aeration under plow tillage further enhances these enzymatic activities by optimizing mitochondrial respiration (Wang et al., 2022). In contrast, no-tillage increases bulk density in the topsoil, restricts root penetration, and reduces the rate of N mineralization due to the development of anaerobic microenvironments. This not only limits root N uptake but also disrupts the balance between N and carbon metabolism, resulting in reduced carbon allocation to starch synthesis (Qin et al., 2006; Fiorini et al., 2018). In addition to protein and starch, maize grains contain considerable amounts of crude fat, essential and non-essential amino acids, and other nutritive components that collectively define their overall nutritional quality. It is hypothesized that plow tillage enhances grain nutritional quality by promoting root elongation and N uptake, and by preferentially allocating the additional N to developing kernels, thereby upregulating protein and starch biosynthesis under full straw return. However, the integrated effects of residue management and varying N application rates on these multifaceted nutritional traits remain insufficiently understood. A major challenge in systems involving full straw return is the absence of a well-defined quantitative N threshold. Without this benchmark, it is difficult to determine an N rate that simultaneously optimizes yield, grain quality, and nutrient use efficiency while avoiding excessive fertilization.

Optimized fertilization under straw returning can balance ecological and economic benefits without compromising yield (Zhang et al., 2025b). Nonetheless, the trade-offs between grain yield, nutritional quality, and NUE under combined residue management and N input remain inadequately characterized. Multi-criteria analytical approaches are increasingly applied to evaluate such complex interactions. Based on a long-term field experiment initiated in 2011, this study was conducted over the 2022 and 2023 growing seasons to assess how residue management and N rates influence maize grain yield, comprehensive nutritional quality index, and NUE. The objectives were to (i) elucidate the effects of these practices on yield, nutritional quality, and NUE, and (ii) determine the optimal N input range that ensures high yield, quality, and efficiency under each crop residue management practice.

## 2 Materials and methods

### 2.1 Experimental site description

**Figure 1:**
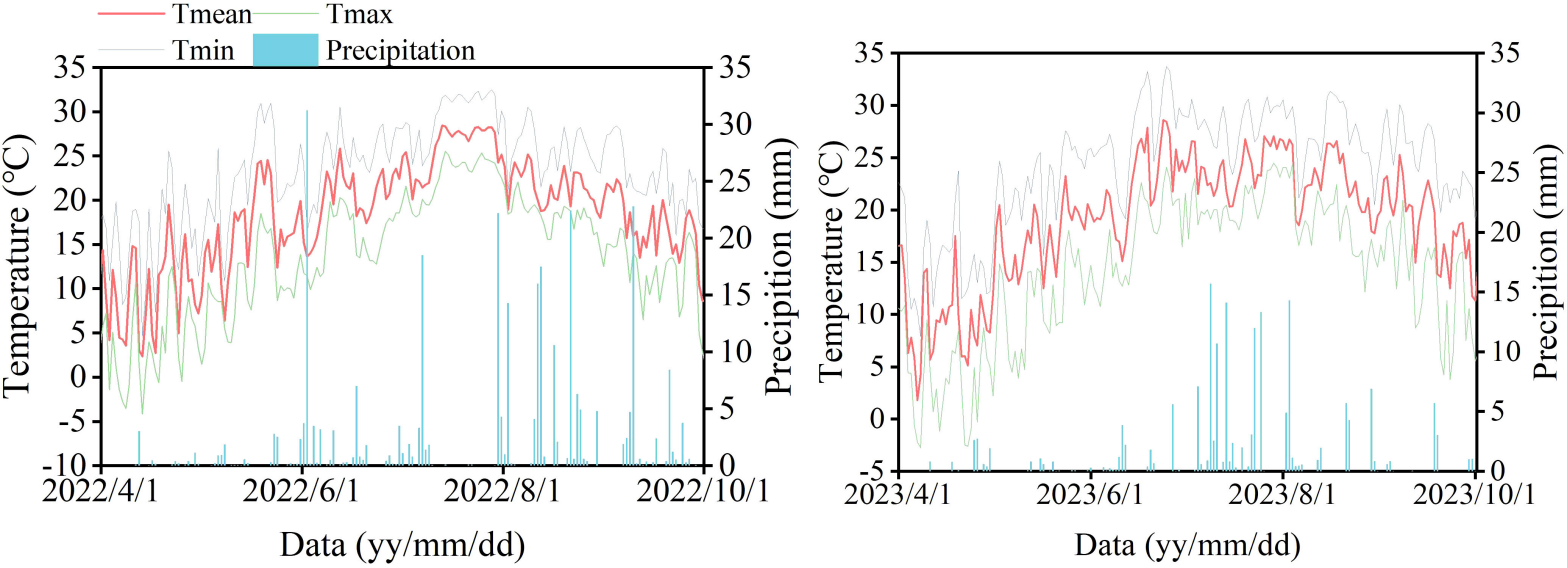
Detailed meteorological information during the maize growth period in 2022 and 2023. Tmean: average temperature (°C); Tmax: maximum temperature (°C); Tmin: minimum temperature (°C).

This long-term field experiment was established in 2011 and conducted at the Halahai Comprehensive Experimental Station of the Jilin Academy of Agricultural Sciences, located in Nong’an County, Jilin Province (44°40′N, 125°07′E). The site experiences a temperate continental monsoon climate, and the soil is classified as Chernozem. The cropping system consisted of continuous maize (*Zea mays* L.) monoculture with full straw return. Prior to sowing in 2012, the physicochemical properties of the 0–20 cm soil layer were as follows: total organic carbon, 1.72 g/kg; total N, 1.20 g kg^−1^; available N, 110.09 mg kg^−1^; available phosphorus, 26.03 mg kg^−1^; and available potassium, 173.98 mg kg^−1^. Meteorological data during the growing season (April–October) are presented in **Figure 1**. The mean temperature was 17.45 °C in 2022 and 12.69 °C in 2023, while total precipitation was 300.35 mm and 304.38 mm, respectively. The soil characteristics under different treatments have been described in detail in previous studies (Meng et al., 2023).

### 2.2 Experimental design

The experiment followed a two-factor factorial design. The first factor involved two crop residue management practices: SM (no tillage with straw mulch, full straw return) and SP (plow tillage incorporating straw mulch to a depth of approximately 15 cm, full straw return). The second factor consisted of five) application rates: N_0_ (0 kg N ha^−1^), N_90_ (90 kg N ha^−1^), N_150_ (150 kg N ha^−1^), N_210_ (210 kg N ha^−1^), and N_270_ (270 kg N ha^−1^). The maize hybrid *Fumin 985* was sown on May 5, 2022, and May 4, 2023. Each treatment plot covered 149.5 m^2^ and was planted at a density of 65,000 plants/ha, with a row spacing of 65 cm and a row length of 23 m. N fertilizer was applied in two stages: 40% as a basal application and 60% as a topdressing. Phosphorus (P_2_O_5_) and potassium (K_2_O) were applied before planting at a rate of 105 kg ha^−1^ each.

### 2.3 Sampling and measurements

#### 2.3.1 Grain dry weight

Thirty uniformly growing plants were tagged at the tasseling stage. Sampling was performed at 10, 20, 30, 40, 50, and 60 days after pollination (DAP). Five ears were collected per treatment at each sampling time. From the central portion of each ear, 1000 kernels were removed and oven-dried at 85 °C until a constant weight was achieved (Zhang et al., 2025a).

#### 2.3.2 Grain yield

At physiological maturity, maize ears were harvested from a 10.4 m^2^ area in each plot to determine ear number per unit area. Twenty ears were selected to count the kernel number per ear. After oven-drying at 85 °C to a constant weight, the 1000-kernel weight was recorded. Grain yield was then calculated on a dry weight basis and standardized to a 14% moisture content, following the method described by Duan et al. (2023).


$$Y=N_{1}N_{2}W/\left(1-0.14\right)\times 10^{-6}$$

where *Y* is the grain yield (Mg/ha), *N_1_* is the number of ears harvested per unit area (ears/ha), *N_2_* is the number of kernels number, and *W* is the 1000-kernel weight (g).

#### 2.3.3 Partial factor productivity of N fertilizer

The partial factor productivity of N fertilizer (*PFPN*), which was the specific proxy used for N use efficiency in this study, was calculated according to the method described by Good et al (Good et al., 2004). The specific calculation formula was as follows:


PFPN=GW/Ns

where *GW* and *Ns* are the grain weight and nutrition supply, respectively.

#### 2.3.4 Grain nutritional quality

The nutritional quality of maize grain, an essential indicator of its nutritive value, was evaluated following the method described by Zhu et al. (2025). Naturally air-dried kernels were analyzed using a near-infrared (NIR) spectrometer (NIRS™ DS3, FOSS, Hillerød, Denmark) within a wavelength range of 780–2526 nm. Based on the distinct absorption and scattering characteristics of major grain components (e.g., crude protein, crude fat, and starch) under near-infrared (NIR) light, spectral data were converted into nutrient concentrations using a maize-specific calibration model. This approach enables rapid and non-destructive quantification of nutritional components. For each grain sample, three replicate NIR spectral measurements were conducted, and the mean value of these three spectra was used as the raw dataset for subsequent analysis.

### 2.4 Statistical analyses

#### 2.4.1 Calculation of coefficient of variation

The coefficient of variation (*CV*) was used to assess the stability of different treatments across varying climatic conditions. The specific calculation formula was as follows:


$$CV=\sigma_{i}/X_{i}\times 100$$

where σ*_i_* and *X_I_* are the standard deviation and mean value of different indicators.

#### 2.4.2 Calculation of comprehensive nutritional quality value

To demonstrate the comprehensive nutritional value of crude protein, starch, crude fat, essential amino acids, and non-essential amino acids, the comprehensive nutritional quality index (Q value) for each treatment was calculated using the entropy weight method, as proposed by Liang et al. (2025), to assess the relative contribution of individual nutritional indicators to overall grain quality. To ensure comparability among indicators with differing magnitudes, all measured grain quality parameters were standardized (*P_ij_*):


$$p_{ij}=\frac{X_{ij}-min\left(X_{ij}\right)}{max\left(X_{ij}\right)-min\left(X_{ij}\right)}$$

The formula for calculating information entropy (*E _j_*) was as follows:


$$E_{j}\;=\;-\frac{1}{\ln30}\times \sum_{i=1}^{30}p_{ij}\times \ln p_{ij}$$

The formula for calculating the entropy weight (*W _j_*) of each index was as follows:


$$W_{j}=\frac{1-E_{j}}{\sum_{j=1}^{5}\left(1-E_{j}\right)}$$

The formula for calculating the Q value (*Y _j_*) of each treatment was as follows:


$$Y_{i}\;=\;\sum_{j=1}^{30}P_{ij}\times W_{j}$$

#### 2.4.3 Variance analysis and data visualization

All· statistical analyses· were executed in R software (v4.3.2). One-way analysis of· variance (ANOVA) was performed using the R package “genstab” (v4.3.2) to evaluate the effects of year, crop residue management, and N application rate on the measured variables. Multi-factor ANOVA was applied to determine the interaction effects among two or more factors. Significant differences between treatments were identified using Fisher’s least significant difference (LSD) test, with statistical significance set at *P* < 0.05. Data visualization and processing were performed using Origin 2021 (OriginLab, Northampton, USA) and Excel 2021 (Microsoft Corporation, Washington, USA).

## 3 Results

### 3.1 Grain growth dynamics

**Figure 2:**
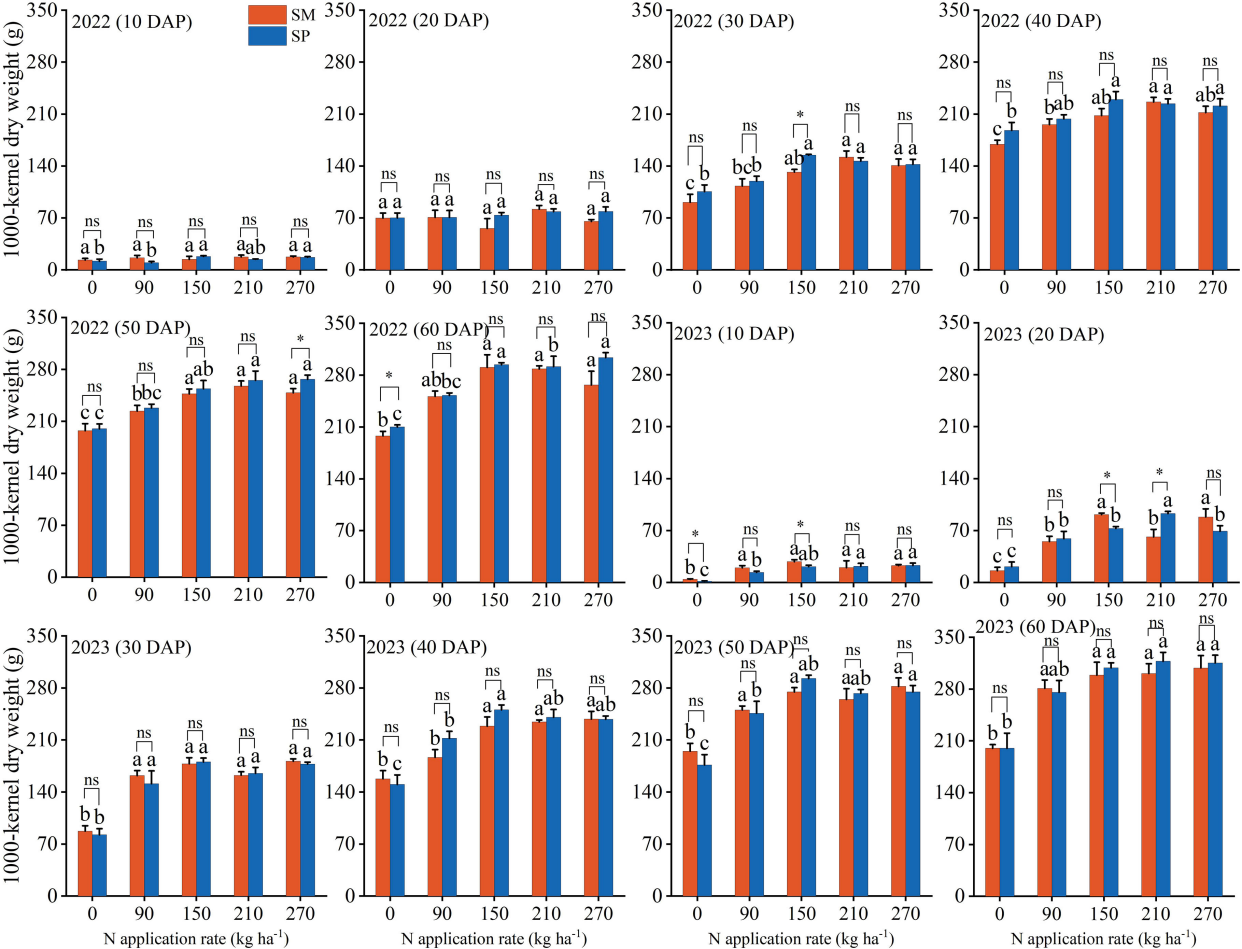
Effects of nitrogen application rate and crop residue management on the dynamic changes in 1000-kernel dry weight after pollination. SM: no tillage with straw mulch; SP: plow tillage with straw mulch. DAP: days after pollination. Different lowercase letters indicate significant differences among nitrogen application rates within the same crop residue management, based on Fisher’s least significant difference (LSD) test (*P* < 0.05). * indicates 0.01 ≤ *P* < 0.05; ** indicates *P* < 0.01; ns indicates *P* ≥ 0.05. The numbers in the figure denote the experimental years. Error bars represent the standard error (SE) based on five biological replicates.

N application rate exerted a significant influence on 1000-kernel weight DAP. Crop residue management (S) and the interaction between S and N significantly affected 1000-kernel weight at 40, 50, and 60 DAP (**Supplementary Table 1**). At 60 DAP, the 1000-kernel weight increased from 202.00 g under N_0_ to 298.69 g under N_270_ across two years. Moreover, at 40, 50, and 60 DAP, the 1000-kernel weight under SP was 4.96%, 1.21%, and 3.18% greater than under SM, respectively. The most pronounced difference was observed at the N_150_ treatment, where SP exceeded SM by 5.66% (**Figure 2**).

The coefficient of variation (CV) in the N0 treatment was the highest among all nitrogen application rates. On average, at 10, 20, and 30 days after pollination (DAP), the CV of 1000-kernel weight under SP was 8.84%, 25.26%, and 9.14% lower than that under SM, respectively. However, the CV of grain yield under SP (9.76%) was slightly higher than that under SM (9.30%) (**Supplementary Table 2**).

### 3.2 Grain yield

**Figure 3:**
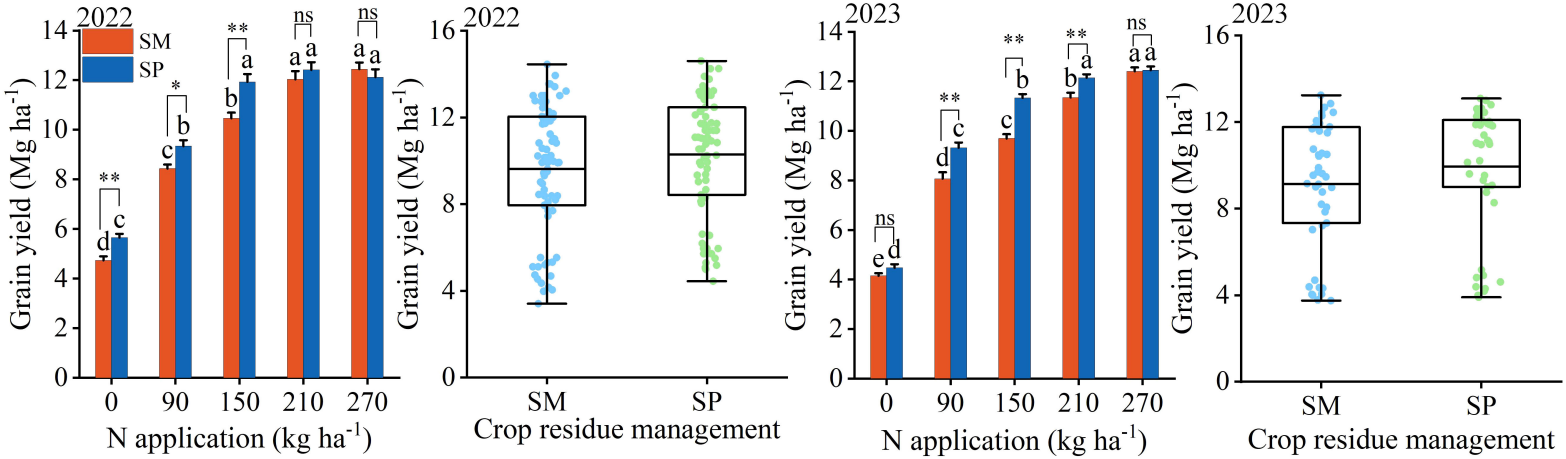
Effects of nitrogen application rate and crop residue management on maize grain yield. SM: no tillage with straw mulch; SP: plow tillage with straw mulch. Different lowercase letters indicate significant differences among nitrogen application rates within the same crop residue management based on Fisher’s least significant difference (LSD) test (*P* < 0.05). * indicates 0.01 ≤ *P* < 0.05; ** indicates *P* < 0.01; ns indicates *P* ≥ 0.05. The numbers in the figure denote the experimental years. Error bars represent the standard error (SE) of five biological replicates.

S, N, and their interaction significantly influenced grain yield (**Supplementary Table 3**). On average, yield increased from 4.75 Mg ha^−1^ at N_0_ to 12.36 Mg ha^−1^ at N_270_. The mean yield under SP was 9.24% higher than that under SM, and this difference was more pronounced at N rates below N_150_, where yield under SP was 14.07% greater (**Figure 3**).

**Figure 4:**
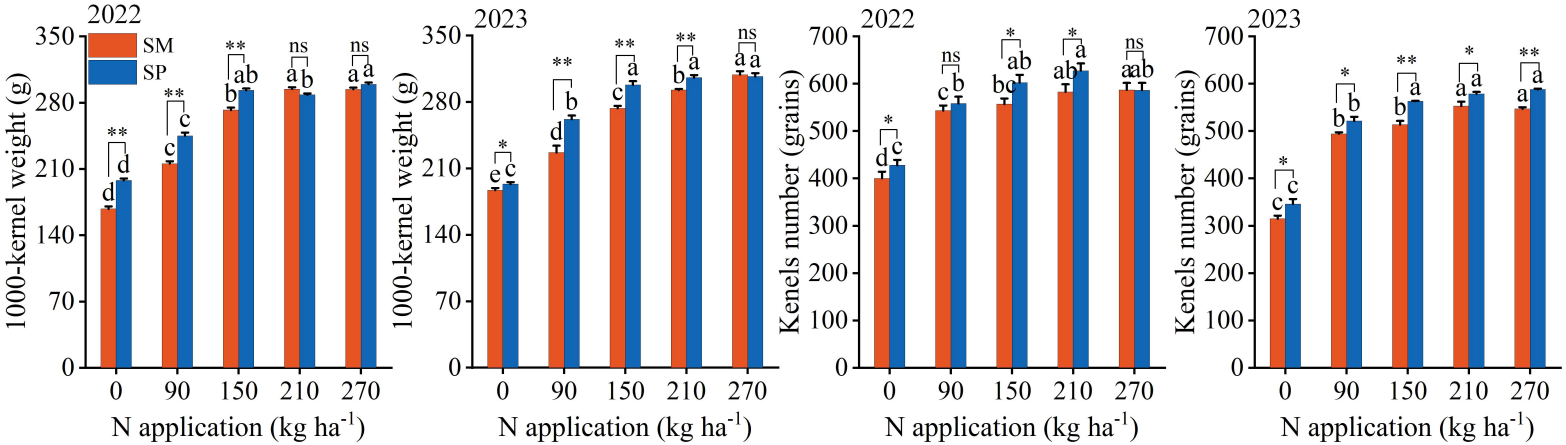
Effects of nitrogen application rate and crop residue management on maize grain yield components. SM: no tillage with straw mulch; SP: plow tillage with straw mulch. Different lowercase letters indicate significant differences among nitrogen application rates within the same crop residue management, based on Fisher’s least significant difference (LSD) test (*P* < 0.05). * indicates 0.01 ≤ *P* < 0.05; ** indicates *P* < 0.01; ns indicates *P* ≥ 0.05. The numbers in the figure denote the experimental years. Error bars represent the standard error (SE) of five biological replicates.

S and N also had significant effects on 1000-kernel weight and kernel number per ear, while their interaction significantly affected only 1000-kernel weight (**Supplementary Table 3**). Over two years, 1000-kernel weight and kernel number increased from 156.56 g and 372.20 kernels per ear at N_0_ to 302.37 g and 577.08 kernels per ear at N_270_, respectively. Furthermore, under SP, 1000-kernel weight and kernel number were 7.09% and 6.26% greater than under SM, respectively. At N rates below N_150_, the advantages of SP became more distinct, with increases of 11.21% in 1000-kernel weight and 7.14% in kernel number compared to SM (**Figure 4**).

### 3.3 Partial factor productivity of N fertilizer

**Figure 5:**
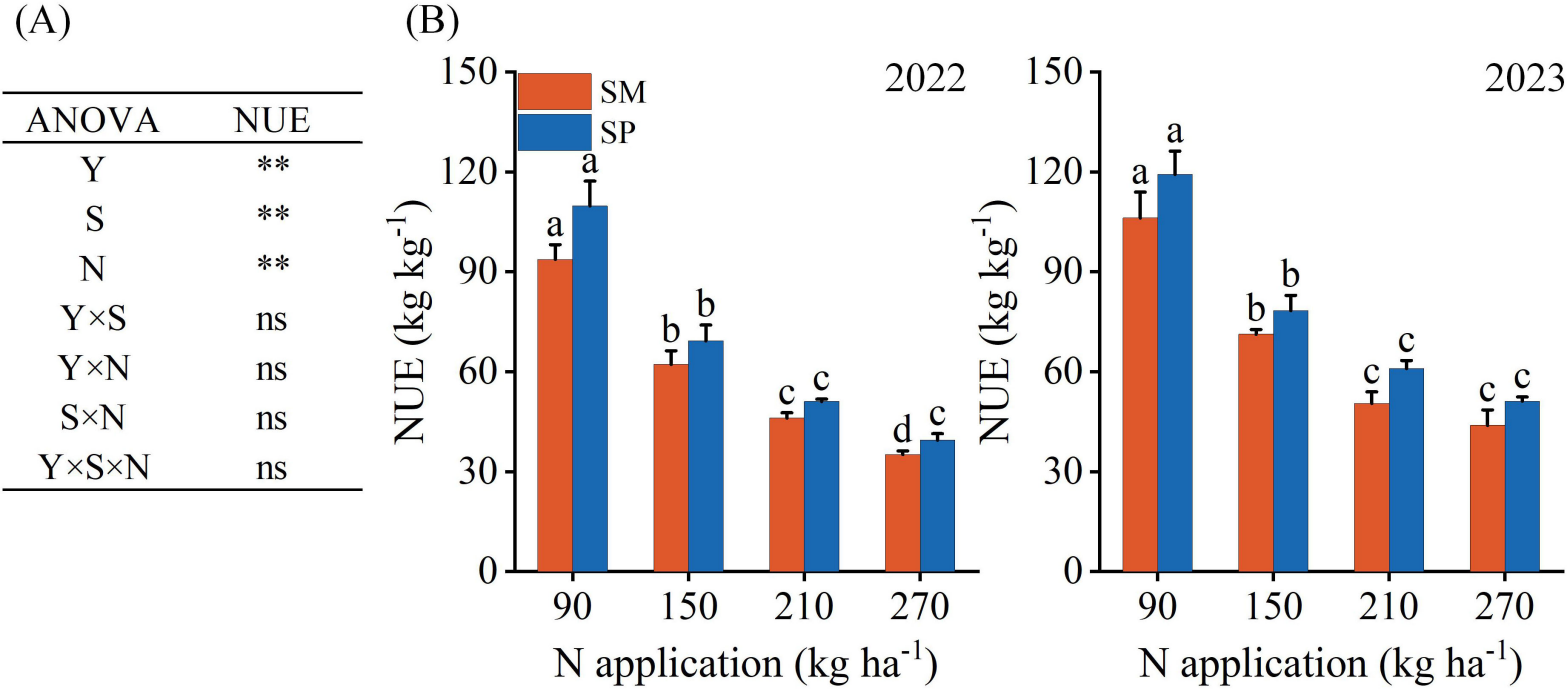
Effects of nitrogen application rate and crop residue management on the partial factor productivity of nitrogen fertilizer (PFPN). **(A)** Variance analysis results; **(B)** PFPN values. SM: no tillage with straw mulch; SP: plow tillage with straw mulch. Y: year; N: nitrogen application rate; S: crop residue management. Different lowercase letters indicate significant differences among nitrogen application rates within the same crop residue management, as determined by Fisher’s least significant difference (LSD) test. (*P* < 0.05). The numbers in the figure denote the experimental years. * indicates 0.01 ≤ *P* < 0.05; ** indicates *P* < 0.01; ns indicates *P* ≥ 0.05. Error bars represent the standard error (SE) of five biological replicates.

S and N had significant effects on the PFPN, while their interaction showed no significant effect (**Figure 5A**). On average, PFPN declined from 107.23 kg kg^−1^ at N_90_ to 42.42 kg kg^−1^ at N_270_. The PFPN under SP was 13.88% higher than under SM (**Figure 5B**), with the greatest difference observed at N_210_, where PFPN was 15.85% higher in SP than in SM.

### 3.4 Grain nutritional quality

**Figure 6:**
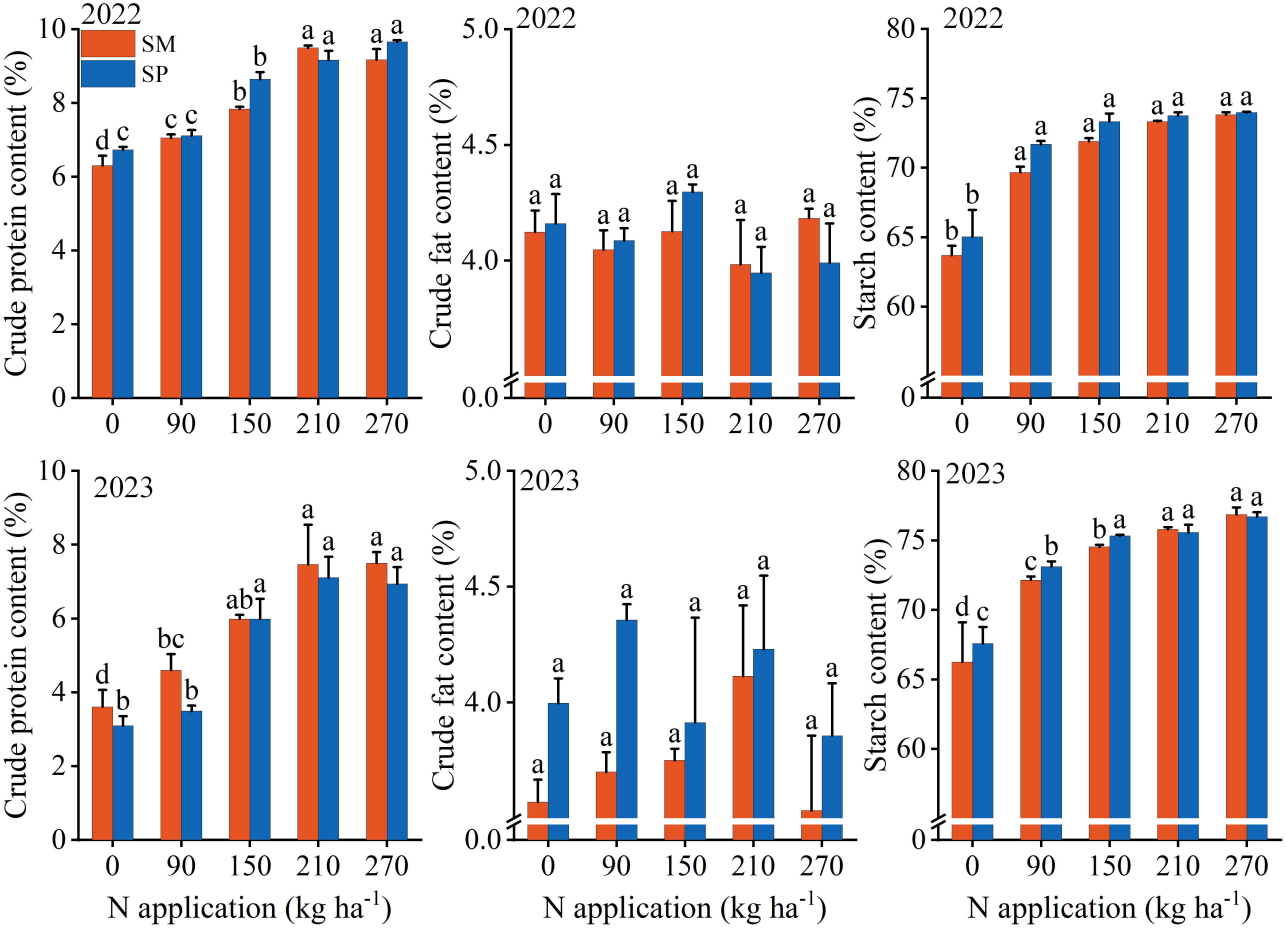
Effects of nitrogen application rate and crop residue management on crude protein, crude fat, and starch contents in maize grains. SM: no tillage with straw mulch; SP: plow tillage with straw mulch. Different lowercase letters indicate significant differences among nitrogen application rates within the same crop residue management, as determined by Fisher’s least significant difference (LSD) test (*P* < 0.05). The numbers in the figure denote the experimental years. Error bars represent the standard error (SE) of three biological replicates.

N significantly affected crude protein and starch contents, while S significantly influenced crude fat and starch contents. The interaction between N and S was not significant for any of the three components (**Supplementary Table 4**). Crude protein content increased from 4.93 mg g^−1^ at N_0_ to 8.31 mg g^−1^ at N_270_, and starch content rose from 65.88% to 75.34% over the same range. Additionally, the crude fat and starch contents under SP were 4.65% and 1.17% higher than those under SM, respectively. At N levels below N_150_, SP enhanced crude fat and starch contents by 6.68% and 1.90%, respectively (**Figure 6**).

**Figure 7:**
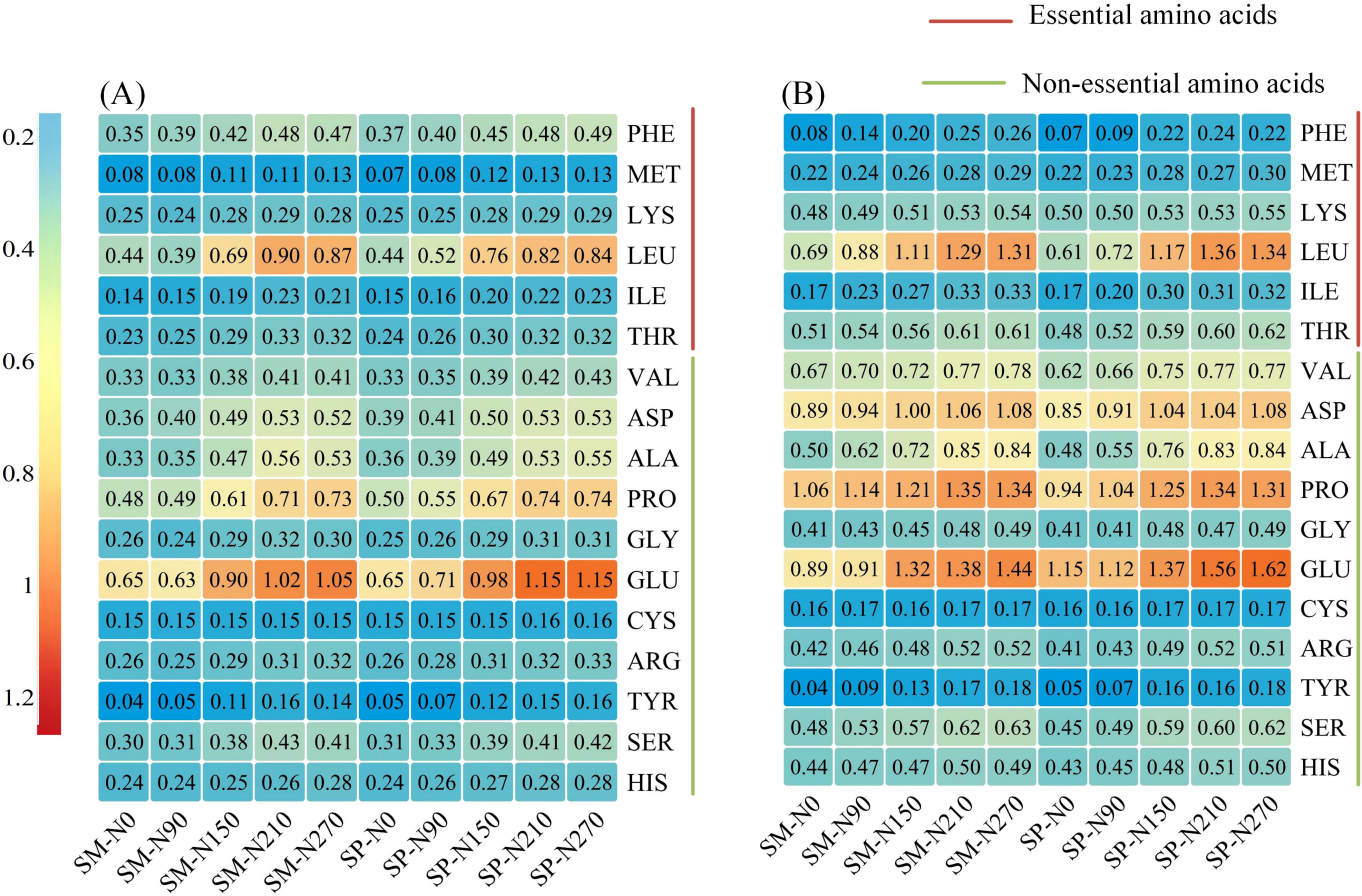
Effects of nitrogen application rate and crop residue management on amino acid contents in maize grains. **(A)** 2022; **(B)** 2023. SM: no tillage with straw mulch; SP: plow tillage with straw mulch. N_0_: 0 kg N/ha; N_90_: 90 kg N/ha; N_150_: 150 kg N/ha; N_210_: 210 kg N/ha; N_270_: 270 kg N/ha. Amino acids include: PHE (phenylalanine), MET (methionine), LYS (lysine), LEU (leucine), ILE (isoleucine), THR (threonine), VAL (valine), ASP (aspartic acid), ALA (alanine), PRO (proline), GLY (glycine), GLU (glutamic acid), CYS (cysteine), ARG (arginine), TYR (tyrosine), SER (serine), and HIS (histidine). The numbers in the figure represent amino acid contents (%).

N also significantly affected total amino acids, essential amino acids, and non-essential amino acids, whereas S and the S×N interaction had no significant effects (**Supplementary Table 5**). Total amino acid content increased from 6.49% at N_0_ to 9.31% at N_270_. Essential and non-essential amino acid contents rose from 1.80% and 4.69% at N_0_ to 2.82% and 6.49% at N_270_, respectively. The relative increase in essential amino acids (12.19%) was greater than that of non-essential amino acids (8.63%) with increasing N application. Amino acids exhibiting increases exceeding 10% included TYR, LEU, ILE, ALA, PHE, and GLU (**Figure 7**).

**Figure 8:**
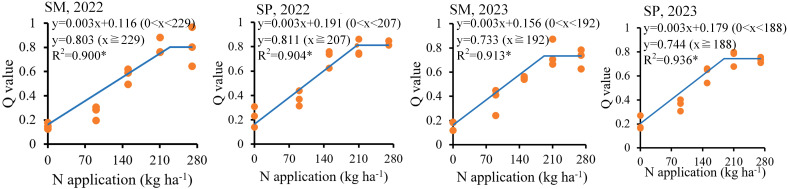
Effects of nitrogen application rate and crop residue management on the comprehensive nutritional quality index (Q value) of maize grains. SM: no tillage with straw mulch; SP: plow tillage with straw mulch. The numbers in the figure denote the experimental years. * indicates *P* < 0.05. R^2^ represents the coefficient of determination.

Regression analysis between N application rate and the comprehensive nutritional quality index (Q value) showed that Q value initially increased and then plateaued with rising N levels (**Figure 8**). The N application rates corresponding to the Q value inflection point were lower for SP (206.67 and 188.33 kg N/ha) than for SM (229.00 and 192.33 kg N/ha) in 2022 and 2023, respectively. Furthermore, the plateau Q value under SP (0.778) was higher than that under SM (0.768).

### 3.5 N application rate balancing yield, PFPN, and Q value

**Figure 9:**
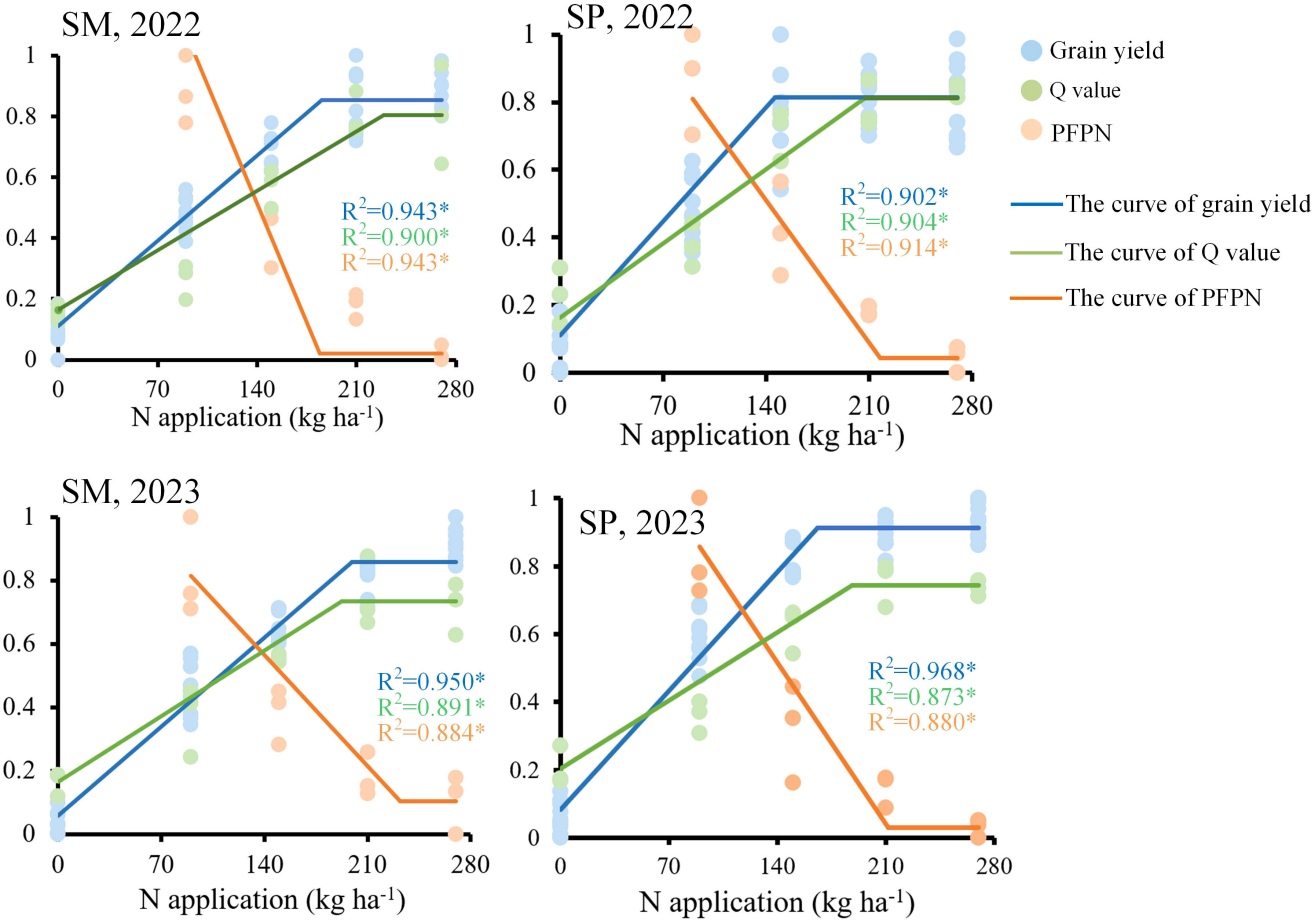
Fitted curves showing the relationships among maize yield, partial factor productivity of nitrogen fertilizer (PFPN), comprehensive nutritional quality index (Q value), and nitrogen application rate. SM: no tillage with straw mulch; SP: plow tillage with straw mulch. The numbers in the figure denote the experimental years. * indicates *P* < 0.05. R^2^ represents the coefficient of determination.

SP required a lower N application rate than SM to reach both yield and Q value plateaus. The plateau N application rates for yield were 192.50 kg N ha^−1^ under SM and 156.10 kg N ha^−1^ under SP. For Q value, the corresponding rates were 210.50 kg N ha^−1^ and 197.00 kg N ha^−1^, respectively, while those for PFPN were 208.26 kg N ha^−1^ and 214.50 kg N ha^−1^ (**Figure 9**). Prior to the plateau stage of the fitted curves, the PFPN curve intersected both the yield and Q value curves. When PFPN was not used as a constraint, an additional N input of 9.35% for SM and 26.20% for SP beyond their respective yield thresholds was required to attain corresponding Q value thresholds. Synergistic improvements in yield, PFPN, and Q value were achieved within N application ranges of 129.90–135.81 kg N ha^−1^ for SM and 113.82–129.53 kg N ha^−1^ for SP. Within these ranges, the maximum grain yield potential reached 67.48%–70.55% for SM and 72.91%–82.97% for SP, while the optimal nutritional potential reached 61.71%–64.50% for SM and 57.78%–65.00% for SP.

## 4 Discussion

### 4.1 Effects of crop residue management and N rate on grain dynamics and nutritional quality of maize

Consumer demand for grain production has progressively shifted from “high yield” to “high yield with superior quality” (Huang et al., 2025). Therefore, achieving both objectives through optimized cultivation techniques represents a fundamental goal of modern agriculture. Insufficient N application suppresses the grain filling rate, active filling duration, and ear differentiation process (Wei et al., 2018; Yu et al., 2021; Wu et al., 2022), thereby reducing grain dry weight and kernel number and ultimately constraining yield improvement (**Figures 2**-**4**). Moreover, N application enhances total starch content in grains by stimulating amylose synthesis (Li et al., 2024) (**Figure 6**). Since starch constitutes approximately 70% of total grain dry matter (He et al., 2021), this increase directly contributes to higher grain dry weight. The observed increase in crude protein content with N application was primarily driven by elevated levels of essential (up by 12.19%) and non-essential amino acids (up by 8.13%) (**Figure 7**), which serve as the structural precursors of proteins (Liu et al., 2024). From a metabolic perspective, higher soil nitrogen availability under SP enhances the activity of nitrogen metabolism enzymes (Yu et al., 2021; Meng et al., 2023; Wang et al., 2024). Glutamine synthetase catalyzes the conversion of ammonium into glutamine in the leaves, which is then transported to the grain as a precursor for storage protein synthesis, thereby contributing to improved grain quality (Lee et al., 2023). Additionally, elevated nitrogen availability under SP upregulates the activity of starch synthase in the endosperm (Gao et al., 2023b), resulting in a 1.90% higher starch content in SP compared to SM (**Figure 6**). Previous studies have shown that excessive N input (e.g., 337 kg N/ha) can reduce starch content by promoting the formation of starch–protein–lipid complexes (Li et al., 2024). In contrast, no decline in starch content was observed in this study at 270 kg N/ha (**Figure 6**), suggesting that this level was below the threshold required to induce substantial complex formation or disrupt starch biosynthesis.

Straw returning enhances crop yield by supplying nutrients and improving soil properties (Wang et al., 2021). Compared with no tillage with straw mulch (SM), plow tillage with straw mulch (SP) increases the straw–soil contact area, stimulates microbial activity, and enhances soil N availability (Zhang et al., 2016). This in turn promotes plant nutrient uptake and biomass accumulation (Li et al., 2024), leading to higher yield potential. The yield advantage of SP was most evident under low N input (< 150 kg ha^−1^), where yield was 14.07% higher than that under SM (**Figure 3**). Furthermore, there were no significant differences in amino acid or protein contents between SM and SP (**Supplementary Table 4** and **S5**). Thus, residue management appears to mainly influence the grain-filling process, resulting in higher starch accumulation under SP than under SM (**Figures 2**, **6**). This difference can be attributed to environmental and soil factors, including physicochemical characteristics, temperature, and precipitation (Gao et al., 2023b).

### 4.2 Influence of crop residue management on N response regulation

As an integral component of nutrient management, straw returning can partially substitute for synthetic N inputs, thereby reducing crop dependence on chemical fertilizers (Yin et al., 2018). However, during straw decomposition, microbial immobilization of N can lead to competition between soil microorganisms and crops, producing variable effects on growth that depend on the residue management strategy (Lin et al., 2024). In this study, the peak yield and grain quality of maize were achieved at lower N application rates under SP. In contrast, SM required an additional 23.32% and 6.58% N input to reach comparable peak yield and quality, respectively (**Figure 9**). This outcome is likely due to SP enhancing available N levels in deeper soil layers (Wang et al., 2025), which improved PFPN by reducing the amount of fertilizer required to achieve equivalent yield (**Figure 5**). Previous research indicates that no tillage systems can substantially increase soil organic carbon content (Kan et al., 2020), but they often maintain lower soil available N compared with plow tillage (Meng et al., 2023). This imbalance in the C/N ratio may intensify competition for N between microorganisms and crops. Moreover, the decomposition of maize straw proceeds more slowly under SM due to elevated soil moisture and lower temperatures prior to planting (Meng et al., 2023). Moreover, differences existed between the two crop residue management practices in synchronizing straw N mineralization with the critical N demand stages of maize. SP improves soil aeration and increases temperature (Meng et al., 2023), thereby accelerating straw decomposition and N mineralization, which aligns nutrient release with crop demand and reduces N deficiency. In contrast, higher soil moisture and bulk density under SM create conditions that slow straw decomposition and delay N mineralization (Zhang et al., 2023). This asynchrony increases the plant’s reliance on nitrogen inputs, explaining why SM requires higher N application rates to achieve comparable yield and quality. The delayed decomposition under SM therefore necessitates greater N input to offset microbial immobilization and sustain optimal yield and quality. Accordingly, N management strategies in systems employing straw return must be carefully adjusted based on the specific residue management practice adopted.

### 4.3 Balanced optimization of yield, nutritional quality, and PFPN

Integrating straw return with N fertilization can effectively enhance both crop yield and PFPN compared with either practice applied independently (Khan et al., 2015). However, improvements in yield and nutritional quality exhibit an optimal response range to N application (**Figure 9**). Beyond this range, excessive N accumulates in the soil due to limited maize uptake (Guo et al., 2024), subsequently decreasing grain PFPN (**Figure 5**). Spatial heterogeneity in optimal N levels arises from variations in residue management, straw incorporation rates, and regional fertilization practices. For instance, in subtropical regions, SP with 200 kg N ha^−1^enhances yield while simultaneously mitigating greenhouse gas emissions (Yang et al., 2022). In Northwest China, straw return combined with 160–180 kg N ha^−1^ increases yield and reduces nitrate leaching risk (Meng et al., 2021). Given the pivotal role of Northeast China in national grain production, optimizing the integration of N fertilization and straw return is crucial. Previous research has shown that SP with 172–254 kg N ha^−1^ and SM with 147–200 kg N ha^−1^ improved soil N retention and yield in the spring maize system of Northeast China (Wang et al., 2025). Additionally, SP with 262 kg N ha^−1^ was identified as optimal for maize starch quality (Li et al., 2024). However, assessing grain nutritional quality solely based on starch is inadequate. Therefore, in this study, a comprehensive nutritional quality index (Q value) incorporating starch, crude fat, crude protein, and amino acid contents was calculated using the entropy weight method to provide an integrated evaluation of maize grain nutritional quality. Our findings revealed that the N application rate required for SP to achieve the synergistic optimization of yield, PFPN, and Q value was 4.62%–12.38% lower than that required for SM (**Figure 9**), demonstrating the superior efficiency of SP in residue management. Nevertheless, although SP saved 42.28–84.97 kg N ha^−1^, it achieved only 72.91%–82.97% of maximum yield potential and 57.78%–65.00% of optimal nutritional quality potential (**Figure 9**). Notably, the identified optimal N thresholds effectively mitigate the typical “dilution effect,” a common trade-off in which increased yield is accompanied by reduced protein or lysine concentrations (Sun et al., 2025). Within the optimal nitrogen range, yield and nutritional quality exhibited a synergistic upward trend (**Figure 9**), with no evidence of declining protein or lysine concentrations (**Figures 6**, **7**). These findings suggest that optimized nitrogen input balances the carbon and nitrogen metabolic demands of maize, thereby avoiding nutrient dilution resulting from excessive yield expansion or inadequate N supply (Lu et al., 2023). This limitation was primarily attributed to PFPN constraints. Future studies could explore increasing planting density as a potential strategy to overcome production limitations imposed by N use efficiency (Guo et al., 2025). Moreover, continuous excessive straw incorporation has been reported to negatively affect crop production (Wang et al., 2021). Therefore, optimizing straw return strategies remains necessary to balance soil fertility improvement with sustainable productivity.

### 4.4 The prospect of crop residue management

While no tillage is widely recognized for its soil health benefits, such as enhanced carbon sequestration and reduced erosion, potential risks associated with long-term no tillage include soil compaction and decreased nitrogen mineralization (Zhang et al., 2023). SP effectively addresses these limitations by disrupting the plow pan, improving soil aeration, and promoting microbial activity (Lu et al., 2023; Zhang et al., 2023). In this study, despite the higher energy and fuel requirements for crop production under SP, the PFPN and grain yield under SP exceeded those under SM by 15.85% and 9.24%, respectively. Moreover, the N application rate required for SP to reach the yield threshold was 29.29% lower than that for SM (Meng et al., 2024). When considering trade-offs among profitability, greenhouse gas emissions, and energy use efficiency, optimizing tillage may be more advantageous than continuous no tillage (Han et al., 2025). Therefore, the sustainability of tillage practices is inherently site-specific. Future research should focus on monitoring the long-term carbon footprint and energy efficiency under SP and SM in Northeast China to further optimize strategies for crop residue and nutrient management. Additionally, only one locally promoted maize cultivar was used in this study, although grain nutritional quality is highly heritable (Yan et al., 2025). Thus, future experiments will include both N-efficient and N-inefficient maize hybrids to verify and refine the identified nitrogen thresholds, thereby enhancing their generalizability. In Northeast China, the recommended N fertilization application rate for maize of 150–240 kg ha^−1^ (Ren et al., 2025). Without considering PFPN, SM and SP could achieve maximum yield while saving 47.55 and 83.90 kg N/ha, respectively, and reach the highest nutritional index while saving 29.50 and 43.00 kg N/ha, respectively. These results indicate that SP enables greater N reduction than SM while maintaining high yield and nutritional quality. Although this study did not assess economic feasibility, developing innovative N management strategies to maximize PFPN remains a key direction for future research. For example, foliar application of nano carbon dots has been reported to enhance grain yield under N-deficient conditions at low cost and with minimal environmental impact (Li et al., 2025a). Overall, this study provides an effective technical reference for achieving high yield, superior nutritional quality, and efficient N utilization in spring maize production systems in Northeast China.

## 5 Conclusion

This study demonstrated the crucial role of crop residue management in optimizing N utilization for spring maize production in Northeast China. SP enhanced dry matter accumulation in kernels during the late grain-filling stage, which increased kernel number and yield while significantly improving starch and fat contents, thereby elevating the overall nutritional quality of maize grain. Based on a multi-objective optimization approach integrating yield, the partial factor productivity of N fertilizer, and nutritional quality, the optimal N application ranges were identified as 113.82–129.53 kg ha^−1^ for SP and 129.90–135.81 kg ha^−1^ for SM under a planting density of 65,000 plants ha^−1^. These optimized ranges provide an important reference for defining N input thresholds that simultaneously ensure high yield, superior grain quality, and efficient nutrient utilization in Northeast China’s maize production systems.

## Data Availability

The raw data supporting the conclusions of this article will be made available by the authors, without undue reservation.
